# Efficient Utilization of Rare Variants for Detection of Disease-Related Genomic Regions

**DOI:** 10.1371/journal.pone.0014288

**Published:** 2010-12-10

**Authors:** Lei Zhang, Yu-Fang Pei, Jian Li, Christopher J. Papasian, Hong-Wen Deng

**Affiliations:** 1 Center of System Biomedical Sciences, University of Shanghai for Science and Technology, Shanghai, People's Republic of China; 2 School of Medicine, University of Missouri-Kansas City, Kansas City, Missouri, United States of America; 3 College of Life Sciences and Engineering, Beijing Jiao Tong University, Beijing, People's Republic of China; Institute of Preventive Medicine, Denmark

## Abstract

When testing association between rare variants and diseases, an efficient analytical approach involves considering a set of variants in a genomic region as the unit of analysis. One factor complicating this approach is that the vast majority of rare variants in practical applications are believed to represent background neutral variation. As a result, analyzing a single set with all variants may not represent a powerful approach. Here, we propose two alternative strategies. In the first, we analyze the subsets of rare variants exhaustively. In the second, we categorize variants selectively into two subsets: one in which variants are overrepresented in cases, and the other in which variants are overrepresented in controls. When the proportion of neutral variants is moderate to large we show, by simulations, that the both proposed strategies improve the statistical power over methods analyzing a single set with total variants. When applied to a real sequencing association study, the proposed methods consistently produce smaller p-values than their competitors. When applied to another real sequencing dataset to study the difference of rare allele distributions between ethnic populations, the proposed methods detect the overrepresentation of variants between the CHB (Chinese Han in Beijing) and YRI (Yoruba people of Ibadan) populations with small p-values. Additional analyses suggest that there is no difference between the CHB and CHD (Chinese Han in Denver) datasets, as expected. Finally, when applied to the CHB and JPT (Japanese people in Tokyo) populations, existing methods fail to detect any difference, while it is detected by the proposed methods in several regions.

## Introduction

Genome-wide association studies (GWAS) have become popular tools for identifying genetic susceptibility variants for complex diseases. The success of this approach relies on the common disease-common variant (CDCV) hypothesis, which presumes that phenotypic variation of common diseases is explained by several common variants, each with a relatively small effect [1], [2], [3]. For the purpose of association mapping, hundreds of thousands of common variants across the genome are genotyped and examined. In cases where causative variants are not directly genotyped, association analysis can still be achieved through indirect linkage disequilibrium (LD) mapping.

Despite the fruitful findings of recent GWAS [4], it is still disappointing that only a small portion of phenotypic variation has been attributed to common, identified genotypic variants for the traits studied. Increasing sample size and/or genotyping greater numbers of SNPs may help identify additional genetic susceptibility variants. However, the potential additional yield of this approach remains to be seen. Extensive studies have provided an alternative to the CDCV hypothesis, termed the common disease-rare variant (CDRV) hypothesis, that may also be important to the etiology of complex diseases [5], [6], [7]. In the CDRV hypothesis, phenotypic variation is assumed to be caused by multiple rare variants [8], [9], [10], [11], [12], [13], [14]. Though individual mutation has a low frequency, their gene-wise or pathway-wise aggregate frequency could be substantially large, which makes it possible for rare variants to be the cause of common diseases. It seems that either the CDCV or CDRV hypotheses hold under certain conditions, and that the etiology of complex disease reflects a mixture of both hypotheses along with effects from other factors, e.g., gene by gene interactions and environments.

When analyzing rare variants for association with phenotypes, ordinary variant by variant methods have insufficient statistical power due to allelic heterogeneity as well as the extreme rarity of individual variants [15]. Alternatively, an efficient strategy has been proposed that considers a set of rare variants in a genomic region as the unit of analysis [15], [16], [17], [18], [19], [20], [21]. One complication that arises in this group-wise strategy, however, is that not all rare variants are potentially disease-causing; rather, a large proportion are believed to represent background neutral variation. Obviously, including rare variants that are irrelevant to phenotype into sets would lead to a reduced signal to noise ratio, with a consequent reduction in statistical power. Recent studies have suggested focusing on non-synonymous variants in gene coding regions [14], [16]. Nonetheless, a remarkable proportion of non-synonymous variants may still represent background population variation [8], [22]. When neutral variants make up a relatively large proportion, analyzing the set with total variants may not represent an optimal approach to performing powerful association tests.

In this study, aiming to improve statistical power of association tests in the presence of neutral rare variants, we propose two alternative strategies. We utilize simulations to investigate and compare the performance of tests with different strategies under a variety of conditions. Finally, we apply the proposed methods to two real sequencing datasets to demonstrate their utilities.

## Materials and Methods

Assume that there are *n_c_* affected individuals and *n_u_* normal individuals, so that the total number of individuals is *n* = *n_c_*+*n_u_*. Assume that a genomic region containing *L* rare variants (MAF<0.01) is to be examined for association with the disease, and genotype data *g_il_* (*i* = 1,…, *n*, *l* = 1,…, *L*) are available at all variants for each individual. Here, the unit of analysis is the set of variants rather than individual variants. Given the set of *L* variants, a variety of grouping strategies exist. In the simplest way, a single set, termed *G_T_*, is formed by including all variants. This grouping strategy, referred to as total grouping, is widely adopted by existing methods. We here describe two alternative grouping strategies. In the first one (selective grouping), two sets are selectively formed: one in which each variant has a greater frequency in cases than in controls, and the other in which each variant has a greater frequency in controls. We term the two formed sets as *G_C_* and *G_U_*. In the second one (exhaustive grouping), a total number of *R* = 2*^L^*-1 subsets (*G*_1_,…, *G_R_*) are formed exhaustively. Below we outline two popular association tests and their implementations with various grouping strategies.

### Collapsing

Given a set of variants, the collapsing method [15] defines an indicator variable for each individual *i*:
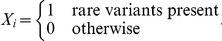



Association is then examined on 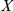 between case and control populations. The authors also proposed a combined multivariate collapsing (CMC) method for analyzing rare and common variants jointly. Since all variants to be investigated in this study are rare, the CMC method is thus not applicable.

In the total grouping strategy, *X* is defined on the set *G_T_*. The Fisher exact test (FET) is used to test the association and a two-sided p-value is reported.

In the selective grouping strategy, *X* is defined and examined on the two sets *G_C_* and *G_U_* separately. To compare with the total grouping method fairly, we perform a two-sided test by examining whether rare variants distribute differently between cases and controls. In order to implement this test, the FET is applied to *G_C_* and *G_U_* separately, and two one-sided p-values, termed *p_c_* and *p_u_*, are obtained. Note that the FET here is merely a device measuring the strength of evidence against the null hypothesis. A provisional index of significance, *q*, is calculated as the minimum of the two nominal p-values obtained
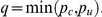



The significance of *q* is determined by Monte Carlo permutation testing [17]. Specifically, we perform *k* replicates. In each replicate, we permute the case/control status and perform the FET on the permuted sample, from which the provisional index for the replicate is obtained. A permutation p-value will be reported, which is defined as *k*_0_/*k*, where *k*_0_ is the number of replicates that have the indexes equal to or smaller than *q*.

In the exhaustive grouping strategy, *X* is defined on each of the *R* formed subsets. The FET is applied to each subset separately, and the smallest two-sided p-value over the *R* tests is taken as the statistic.

Identifying the subset of variants that renders the smallest p-value by testing subsets exhaustively, however, is computationally demanding. When the number of variants involved is large, e.g., several dozens to hundreds, the exhaustive grouping strategy may become computationally prohibitive. We note that some subsets do not need to test. Taking a particular set *G* as an example, if one of its subsets *G*_-*r*_, formed by removing a variant *r* from *G*, renders a smaller p-value than that attained with *G*, then none of subsets of *G* containing the variant *r* are likely to yield a smaller p-value than attained with *G*_-*r*_, and consequently do not need to test. This observation is driven by the fact that all variants in a set act additively. We thus propose a simple and efficient algorithm to reduce the number of tests. The algorithm is outlined below:

1. Set a candidate global set of variants *G*, which is specified by the rare variants it contains, and is initialized to include all variants. Set a global variable *P* for the minimal p-value, which is initialized to be equal to the p-value rendered by the initialized set *G*.
2. For each variant in *G*, remove it from *G* to form a subset *G*_−1_. Collapsing test is then examined on *G*_−1_ and a two-sided p-value is obtained. For *m* rare variants in *G*, there will be *m* subsets each consisting of *m*-1 rare variants. Of the *m* calculated p-values, the minimal one and the corresponding subset are denoted as *P*' and *G_min_*, respectively.
3. Set *G* = *G_min_*, and set *P* =  *P*' if *P*'<*P*.
4. Repeat steps 2) and 3) until the set *G* contains no variant.

The value *P* will be the p-value sought. Here, we adopt a step-wise approach and exclude one variant in each repetition. In this way, the number of subsets that need to be tested is reduced from 2*^L^*-1 to *L*(*L*+1)/2 and the computation cost is reduced accordingly. By running 100 simulated datasets each with 10 or 20 variants, we observed that the algorithm found the correct minimal p-values in all cases. Nonetheless, there is no guarantee for the accuracy, so we will call this test the approximately optimal collapsing (AOC).

The significance of the test is again evaluated by Monte Carlo permutation testing. Specifically, the case/control status is permuted *k* times, and for each replicate the approximately smallest p-value is calculated by the algorithm. A permutation p-value is estimated and reported.

### Group-wise weighted sum test (GWWS)

GWWS performs a one-sided test of whether there is an excess of rare variants in a particular population, i.e., cases. In the total grouping strategy, the GWWS method [17] is comprised of the following steps for testing association:

1. For each variant *l*, calculate a weight 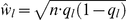, where 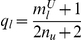, where 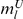 is the number of mutant allele observed in the control population.
2. For each individual *i*, calculate a genetic score 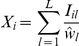, where *I_il_* is the number of mutant allele at the *l*th variant.
3. All individuals are ranked according to genetic scores calculated in 2).
4. The sum of rank of case individuals, termed *S*, is taken as the statistic.

The significance of the test is again evaluated by permutation testing. Specifically, in each of *k* replicates, case/control status is permuted and the statistic is calculated. A normality approximation is used to estimate p-value. Denote the mean and standard deviation of *S* over the *k* replicates as 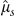 and 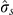. A normalized statistic is given by
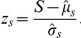



The statistic *z_s_* approximately follows a standard normal distribution under the null hypothesis of no association, from which a two-sided p-value will be evaluated and reported.

In the selective grouping strategy, GWWS is applied to *G_C_* and *G_U_* separately, and two normality approximated p-values, *p*_1_ and *p*_2_, are obtained. The final p-value *p_s_* is taken as the minimal of the two p-values adjusted by the Bonferroni correction
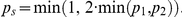



The exhaustive grouping strategy is applied to GWWS as well in principle. However, the computation will be expensive since for each of the *R* subsets, a series of permutations are required to evaluate the significance. We thus do not implement the exhaustive grouping strategy for GWWS.

There also exist several extensive methods that test association between rare variants and diseases [14], [18]. As the purpose of the current study is to compare different grouping strategies rather than different tests, we thus do not include these extensive methods into analysis.

### Simulation studies

#### Simulating allele frequency spectrum

In order to investigate and compare the performance of various methods, we conducted a series of simulation studies. We focused on the European population and simulated sequence data by the use of a four-parameter demographic model [16], [23]. In this model, the shape of European population history is assumed to start by a constant ancestral population, followed by a population bottleneck with a reduction in effective size, and then by an exponentially expansion of the population until to the present. The four parameters involved are the constant ancestral population size *N*_1_, the bottleneck population size *N_b_*, the duration of time *T* after the bottleneck (measured by generation), and the population growth rate 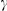 after the bottleneck. The detailed inference of the model was described previously [16], [24], and in the appendix S1, we outlined the process. We formulated the demographic model in a likelihood framework, and estimated the parameters of the model by analyzing the real sequencing data produced by the ENCODE3 project [25]. The sequence data simulated from the model fitted the real data well (Figure S1).

Using the inferred demographic model, we simulated full range frequencies of sequence data to mimic a sequencing association study. Specifically, we simulated sequence data for a gene with 1,000 nucleotides. The number of generated variants may vary, but is approximately proportional to gene length.

#### Simulating phenotype

Both common and rare variants may contribute to complex diseases. However, the usual way for an association study is to analyze these two kinds of variants separately. As the intention of this study is to analyze rare variants, we thus use rare variants (MAF<1%) only to generate phenotype and to test association. Though we focused on a case/control study design, we simulated a pool of quantitatively phenotyped individuals, from which we selected individuals with extreme phenotypes as case/control subjects. This extreme sampling study design is widely adopted in practical applications [10], [14], [16], and its advantage over conventional sampling according to case/control status is that enlarging the pool of phenotyped individuals alone can increase statistical power to detect association, without additional sequencing cost. Specifically, we simulated a pool of 50,000 phenotyped individuals, from which 500 individuals with the lowest phenotypes and 500 with the highest were selected and labeled as control and case subjects, respectively.

On simulating phenotype, a specified proportion of variants were randomly selected as causative, while the remainder were assumed to be neutral. Unless otherwise specified, we set the proportion to be equal to 30%. To take into account the uncertainty of phenotypic model under which multiple rare variants jointly influence phenotype, we simulated the phenotype under three phenotypic models.

Model 1: Causative alleles affected the phenotype equally and in a cumulative way. Specifically, we defined a genetic score for each individual *i*

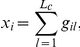

where *L_c_* was the number of causative variants. Since homozygous mutant genotypes are much rarer for rare variants, we assumed an additive mode of inheritance and encoded 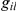 as 0, 1, or 2 for homozygous wild type, heterozygous type, or homozygous mutant genotypes.
Model 2: The presence of one or more causative variants caused the same shift of phenotypic mean. Specifically, the genetic score was defined as an indicator
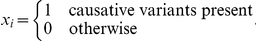

This model corresponds to the assumption adopted by the collapsing method [15].
Model 3: Rarer variants were assumed to render larger per-allele effects. Correspondingly, the genetic score was defined as a weighted sum
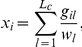

where 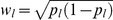, where *p_l_* was the allele frequency in the population. This model corresponded to the assumption adopted by GWWS [17].

In table S1, we list the interaction of various phenotypic models and genotypic encoding schemes by various tests. Note that other intermediate phenotypic models could also be the case for the trait, which will not be investigated in this study.

Under each model, the phenotype was generated by the ordinary regression equation
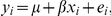



where *μ* was the grand mean; *e_i_* was assumed to follow a normal distribution with mean zero. The coefficient *β* was determined by the locus heritability *h* ^2^, which was defined as the proportion of phenotypic variation explained by *x*,
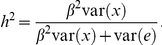



#### Evaluating performance

We evaluated the performance of various tests described in this manuscript: collapsing, GWWS, approximately optimal collapsing (AOC), selective collapsing (SCollapsing), and selective GWWS (SGWWS). Statistical properties, including type I error rate and power, of these tests were investigated by simulation studies. Type I error rate was evaluated by setting the locus heritability *h* ^2^ to zero, whereas power was evaluated by setting it to a specific value. Unless otherwise specified, the value of *h* ^2^ was set to 0.5% to simulate a modest genetic effect. Both type I error rate and power were estimated on 1,000 replicates, at the significance levels of 0.05 and 1.0E-3, respectively. For each replicate, 10,000 permutations will be performed when needed. Both type I error rate and power were defined as the proportion of replicates in which the p-value was equal to or smaller than the significance threshold.

### Simulation results

#### Type I error rates

While all other tests have correct type I error rates that are close to the target level, SGWWS has a type I error rate lower than the target level (table 1). This conservative performance is caused by the fact that the Bonferroni correction is used to adjust two dependent tests in SGWWS. We also estimated type I error rates on naturally occurred sequence data of Chinese Han in Beijing (CHB) and Yoruba people of Ibadan (YRI) populations of the ENCODE3 project. To accomplish this, we permuted all individuals' population attribute, and then analyzed the permuted datasets. Again, SGWWS has a conservative performance while other tests are robust in all studied genomic regions (table 2).

**Table 1:** Type I error rates.

		Gene length			
Sequenced size	Test	500	1,000	1,500	2,000
500	Collapsing	3.4	4.0	3.8	3.9
	SCollapsing	4.8	4.9	4.9	4.6
	AOC	5.1	4.9	5.0	5.2
	GWWS	5.1	5.1	4.8	4.8
	SGWWS	2.6	3.6	3.8	4.2
1,000	Collapsing	3.8	4.7	4.3	4.1
	SCollapsing	4.6	4.9	5.2	4.9
	AOC	4.7	5.1	5.2	4.9
	GWWS	4.9	5.0	5.0	5.5
	SGWWS	2.7	3.5	4.1	4.4
2,000	Collapsing	4.3	4.7	4.4	4.0
	SCollapsing	4.8	5.3	4.7	5.1
	AOC	4.9	5.0	4.8	5.2
	GWWS	5.0	5.0	5.1	4.9
	SGWWS	2.8	3.7	4.0	4.3

Notes: A total number of 50,000 quantitatively phenotyped individuals were simulated, of which 500, 1,000, and 2,000 individuals with equally numbers of highest and lowest phenotypes were selected as case and control subjects. The gene length varied from 500 to 2,000 nucleotides. Type I error rates were estimated on 1,000 replicates at the significance level 0.05. For each replicate, 10,000 permutations were performed. Abbreviation: Collapsing, the collapsing method proposed by Li and Leal [15]; SCollapsing, the collapsing method with selective grouping strategy; AOC, the approximately optimal collapsing; GWWS, the group-wise weighted sum method proposed by Madsen and Browning [17]; SGWWS, the GWWS method with selective grouping strategy.

**Table 2:** Type I error rates on the ENCODE3 datasets.

		Test				
Region	#variants	Collapsing	SCollapsing	AOC	GWWS	SGWWS
ENr221	90	4.8	**5.0**	**5.1**	5.1	**3.9**
ENm010	152	3.9	**4.8**	**4.9**	4.9	**4.1**
ENr321	141	4.8	**5.1**	**5.0**	4.8	**4.0**
ENr232	127	5.1	**4.9**	**4.8**	4.5	**4.6**
ENr123	76	4.7	**4.9**	**5.2**	4.8	**4.5**
ENr213	126	5.2	**5.2**	**4.6**	5.1	**3.9**
ENr133	102	4.7	**4.6**	**4.8**	5.2	**4.5**

Notes: Seven ENCODE3 regions are available for analysis. In each region, we tested the overrepresentation of rare variants between the CHB (90 individuals) and the YRI populations (120 individuals). The MAF cutoff 1% was applied. For each dataset, the total number of variants was given. To evaluate the type I error rate, we permuted each individual's population attribute and analyzed the permuted datasets. Type I error rates were estimated on 1,000 replicates at the significance level 0.05. See notes for table 1 for abbreviation detail.

#### Power with the proportion of causative variants

**Figure 1:**
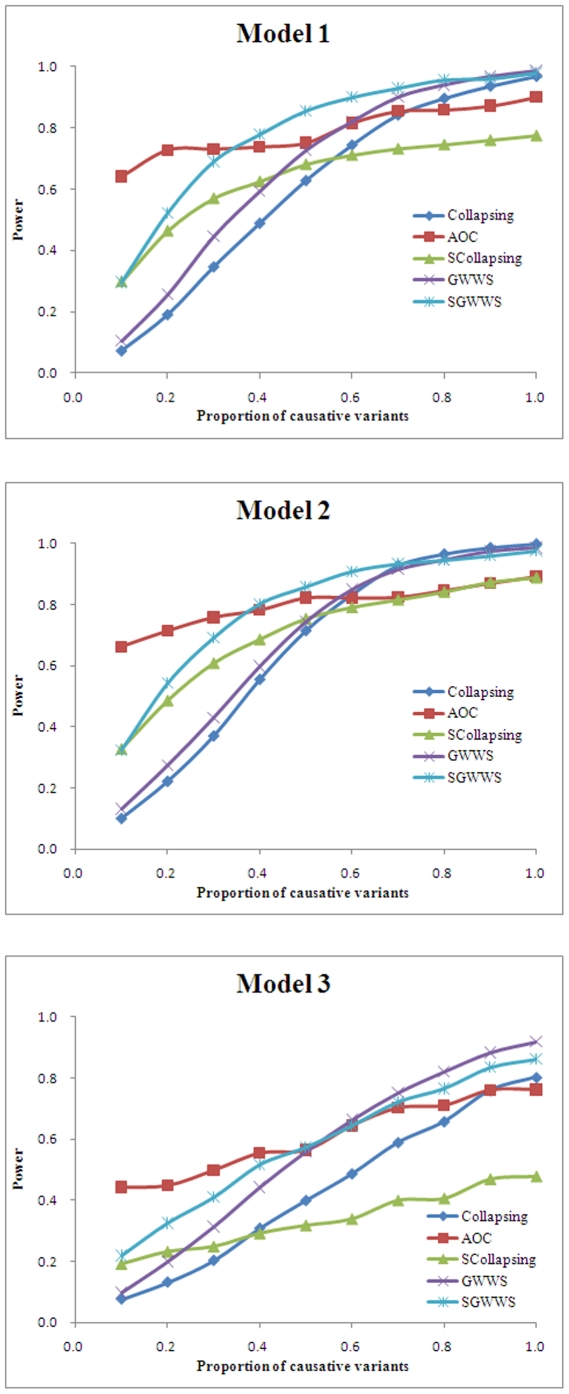
Power with the proportion of causative variants. A total of 50,000 phenotyped individuals were simulated, of which 500 with lowest and 500 with highest phenotypes were selected as control and case subjects, respectively. A gene with 1,000 nucleotides was simulated, and the proportion of causative variants varied from 0.1 to 1.0. The gene was assumed to explain a proportion of 0.5% of phenotypic variation. Three phenotypic models were simulated. In model 1, causative alleles affected the phenotype equally and in a cumulative way. In model 2, the presence of one or more causative variants caused the same shift of phenotypic mean. In model 3, rarer causative variants had a larger per-allele effect and variants contributed to the phenotype in a cumulative way. Power was estimated on 1,000 replicates at the significance level 1.0E-3.

In figure 1, we estimated the power of various tests under different proportions of causative variants. Obviously, all tests have improved power with increasing proportion of causative variants. At low proportion of causative variants, tests that are based on alternative grouping strategies have largely improved power over tests on grouping all variants. For example, at proportion 0.1, the power of AOC, SCollapsing and SGWWS under model 1 are 64.1%, 29.8% and 29.7%, while that of Collapsing and GWWS are only 7.4% and 10.5%, respectively. When the proportion increases, the magnitude of power improvement gets minor. Finally when at high proportion, tests with grouping all variants instead have higher power. This simulation clearly shows that when the proportion of causative variants is low to modest, the proposed alternative grouping strategies are advantageous.

#### Power with gene heritability

**Figure 2:**
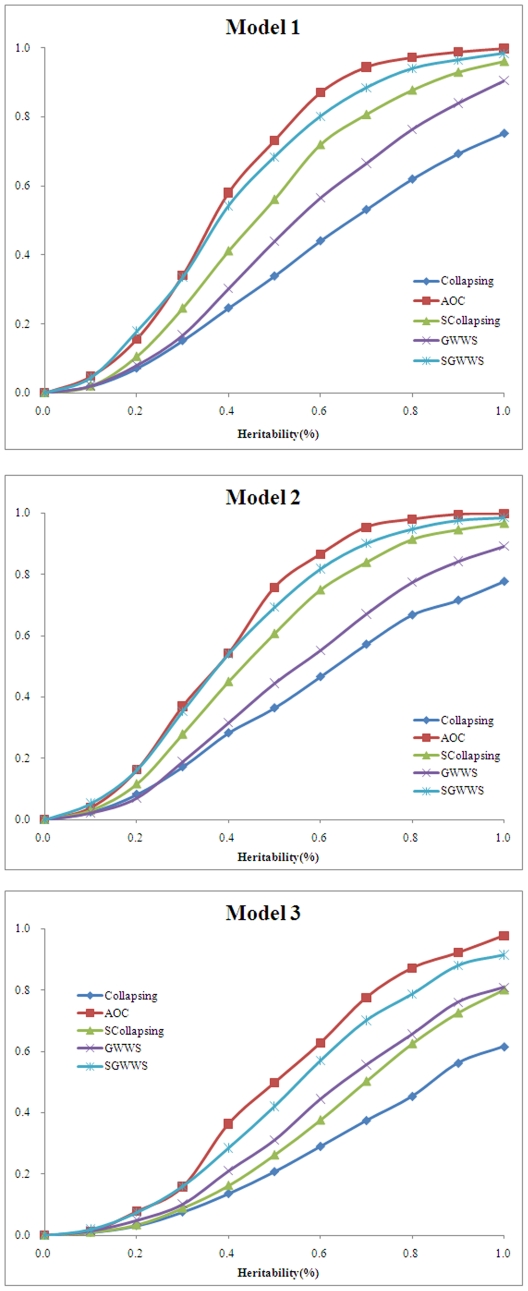
Power with gene heritability. A total of 50,000 phenotyped individuals were simulated, of which 500 with lowest and 500 with highest phenotypes were selected as control and case subjects, respectively. A gene with 1,000 nucleotides was simulated, and the proportion of causative variants was set to 30%. Three phenotypic models were simulated. Under each model, the gene heritability varied from 0.1% to 1.0%. Power was estimated on 1,000 replicates at the significance level 1.0E-3.

We then evaluated the power of various tests under different gene heritabilities. All tests have improved power with increasing heritability (figure 2). Among tests, AOC has the highest power, followed by SGWWS, SCollapsing, GWWS, and Collapsing. Phenotypic model has limited influence in relative performance of tests, but all tests have lower power in model 3 than in models 1 and 2. AOC, SGWWS, and SCollapsing have power around 75%, 70% and 60% respectively to detect gene with 0.5% heritability in models 1 and 2, while that of GWWS and Collapsing are 45% and 35%. When the heritability finally increases to 1.0%, tests with alternative groupings have nearly 100% power in models 1 and 2, and over 80% in model 3. GWWS has approximately 90% power in models 1 and 2, and in model 3 its power is around 80%. While the power of Collapsing in models 1 and 2 maintains at 75%, it is only 60% in model 3.

#### Power with gene length

**Figure 3:**
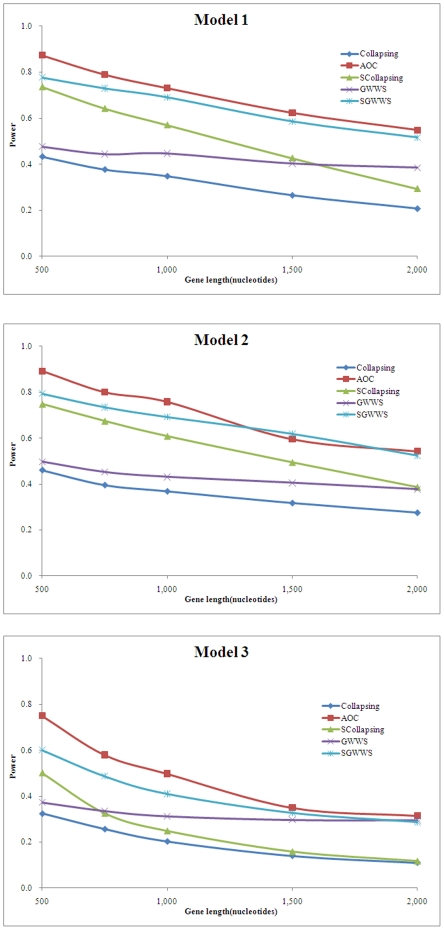
Power with gene length. A total of 50,000 phenotyped individuals were simulated, of which 500 with lowest and 500 with highest phenotypes were selected as control and case subjects, respectively. The proportion of causative variants was set to 30%, and the gene heritability was set to 0.5%. Three phenotypic models were simulated. Under each model, the gene length varied from 500 to 2,000 nucleotides. Power was estimated on 1,000 replicates at the significance level 1.0E-3.

Fixing the proportion of causative variants and gene heritability, we then estimated the effect of gene length on power estimate. As shown in figure 3, all tests have decreased power with increased gene length in all phenotypic models. Among tests, tests with alternative grouping strategies generally have higher power over GWWS and Collapsing.

#### Power with phenotyped sample size

Table 3 lists the power of various tests with different numbers of phenotyped individuals. Obviously, enlarging the pool of phenotyped individuals alone can substantially improve the power. Again, tests with alternative groupings are more powerful than that with grouping all variants.

**Table 3:** Power with phenotyed sample sizes.

		Phenotyped Sample Size		
Phenotypic Model	Test	25,000	50,000	100,000
Model 1	Collapsing	0.23	0.35	0.45
	SCollapsing	0.42	0.57	0.72
	AOC	0.64	0.73	0.85
	GWWS	0.30	0.45	0.56
	SGWWS	0.54	0.69	0.81
Model 2	Collapsing	0.28	0.37	0.46
	SCollapsing	0.45	0.61	0.73
	AOC	0.56	0.76	0.85
	GWWS	0.30	0.43	0.54
	SGWWS	0.55	0.69	0.80
Model 3	Collapsing	0.14	0.20	0.29
	SCollapsing	0.17	0.25	0.39
	AOC	0.34	0.50	0.69
	GWWS	0.20	0.31	0.44
	SGWWS	0.30	0.41	0.58

Notes: The number of phenotyped individuals varied from 25,000 to 100,000. A gene with 1,000 nucleotides was simulated, and three phenotypic models were considered. Thirty percent of the produced variants were assumed to be causative. The gene was assumed to explain 0.5% of the total phenotypic variation. Power was estimated on 1,000 replicates at the significance level 1.0E-3. See notes for table 1 for abbreviation detail.

#### Computation time

The computation time was determined by running a dataset containing 500/500 individuals and a gene with 1,000 nucleotides on a desktop computer with an Intel 2.40 GHz Core 2 Duo CPU E4600. All tests but AOC complete the computation within seconds for 10,000 permutations; AOC takes approximately one hour with 1,000 permutations. This is because the time complexity for AOC is quadratic to the number of variants while it is linear for other tests.

### Application I

As a first application, we re-analyzed the sequence data produced by Cohen et al. [11]. In their study, in order to examine the association of the candidate gene *NPC1L1* with low density lipoprotein cholesterol (LDL-C) level, the authors sequenced the gene in an initial sample with two groups each comprising 128 individuals with lowest and highest ratios of campesterol to lathosterol (Ca∶L ratio) from the Dallas Heart Study (DHS, Sample 1). A total number of 16 rare non-synonymous variants were discovered, 13 of which are exclusive in cases and 3 exclusive in controls.

To validate their findings, the authors sequenced the same gene in a second sample comprising the same numbers of individuals again from the DHS with the next lowest and highest Ca∶L ratios (Sample 2). A total number of 12 rare non-synonymous variants were discovered, 10 of which are exclusive in cases and 2 exclusive in controls.

In addition to analyzing the two samples separately, we combined them and analyzed the total rare variants together (Combined).

### Application I results

As shown in table 4, all tests have the ability to detect the association at the significance level 0.05, but tests with the proposed grouping strategies produce smaller p-values than those with grouping all variants. For example, when applied to the combined sample, AOC, SCollapsing, and SGWWS have p-values 3.90E-5, 3.68E-5, and 5.86E-6, while that of Collapsing and GWWS are 1.23E-4 and 1.00E-4, respectively.

**Table 4:** Analyses of the sequence data produced by Cohen et al. [11].

	Test				
Sample	Collapsing	SCollapsing	AOC	GWWS	SGWWS
Sample 1	3.16E-3	**1.29E-3**	**1.60E-3**	1.96E-3	**4.31E-4**
Sample 2	0.03	**0.01**	**0.02**	0.02	**0.02**
Combined	1.23E-4	**3.68E-5**	**3.90E-5**	1.00E-4	**5.86E-6**

Notes: The candidate gene *NPC1L1* was sequenced in two samples. The first sample (Sample 1) was comprised of 128 individuals with highest ratios of campesterol to lathosterol (Ca∶L ratio) and 128 individuals with lowest ratios from the Dallas Heart Study (DHS). A total number of 16 non-synonymous variants were analyzed, 13 of which are exclusive in cases and 3 exclusive in controls. The second sample (Sample 2) was comprised of the same number of individuals with the next highest and lowest Ca∶L ratios from the DHS. A total number of 12 non-synonymous variants were analyzed, 10 of which are exclusive in cases and 2 exclusive in controls. The combined sample was formed by combining the two samples together. See notes for table 1 for abbreviation detail.

### Application II

As a second application, we analyzed the sequence datasets produced by the ENCODE3 project [25]. In the ENCODE3 project ten 100 kb genomic regions were sequenced in 11 populations. We selected four populations: CHB, Japanese people in Tokyo (JPT), Chinese Han in Denver (CHD), and YRI for analysis, of which 90, 97, 30, and 120 individuals were sequenced respectively. Data on seven genomic regions were available for analysis. Genotype data (released on March 14^th^ 2008) were downloaded from the project ftp site (ftp://ftp.hgsc.bcm.tmc.edu/pub/data/HapMap3-ENCODE/ENCODE3//ENCODE3v1/). Variants that failed to be called in all individuals were excluded. Within each region, we examined the difference of rare allelic distributions between CHB and each of the other three populations. This is equivalent to an association study by labeling individuals from one population as cases and that from the other population as controls [17]. A number of 10,000,000 permutations were replicated. Since GWWS, unlike the other encoding schemes, can only test whether there is an enrichment of rare variants in a particular population, we perform the test twice and report the minimal of the two p-values.

### Application II results

We first tested the overrepresentation of rare variants between the CHB and YRI populations. The results from the proposed tests clearly show that rare allelic distribution between the two populations is significantly different (table 5). Among the tests, SCollapsing produces the smallest p-values, followed by AOC and SGWWS, and GWWS, and at last Collapsing. At some regions, p-values produced by the proposed tests are extremely small. For example, when analyzing the region ENr213, SCollapsing, AOC, and SGWWS have p-values 1.00E-7, 1.00E-7, and 1.22E-7, while that of GWWS and Collapsing are 3.72E-4 and 1.27E-3. Though GWWS and Collapsing have the ability to detect the difference in some regions, in other regions their p-values are not significant even at the level 0.05.

**Table 5:** Analyses of the ENCODE3 datasets for the CHB and YRI populations.

		Test				
Region	#variants	Collapsing	SCollapsing	AOC	GWWS	SGWWS
ENr221	90(31/59)	0.11	**5.37E-6**	**9.00E-4**	0.02	**1.53E-4**
ENm010	152(56/96)	0.58	**2.21E-6**	**1.10E-3**	0.04	**1.22E-3**
ENr321	141(53/88)	0.20	**2.04E-7**	**1.00E-7**	0.02	**6.25E-6**
ENr232	127(59/68)	0.89	**6.63E-4**	**0.01**	0.34	**0.03**
ENr123	76(27/49)	1.00	**8.10E-4**	**0.02**	0.50	**0.03**
ENr213	126(35/91)	1.27E-3	**1.00E-7**	**1.00E-7**	3.72E-4	**1.22E-7**
ENr133	102(37/65)	0.03	**1.63E-5**	**8.05E-4**	0.03	**1.98E-4**

Notes: We tested the overrepresentation of rare variants between the CHB (90 individuals) and YRI populations (120 individuals). For each region, the total number of variants was given, followed by the number of variants that have larger frequencies in the CHB population (the first number in the parenthesis) and the number of variants that have larger frequencies in the YRI population (the second number in the parenthesis). See notes for table 1 for abbreviation detail.

We then analyzed the CHB and JPT populations, as listed in table 6. Collapsing and GWWS report none regions to exhibit difference at the significance level 0.05. In contrast, SCollapsing detects four regions that have different distributions between the two populations. Taking two additional regions reported by SGWWS into account, of 7 regions there are 6 are detected by the proposed tests to exhibit difference. These findings clearly demonstrate the ability of the proposed tests in detecting tiny effects that the existing methods fail to detect.

**Table 6:** Analyses of the ENCODE3 datasets for the CHB and JPT populations.

		Test				
Region	#variants	Collapsing	SCollapsing	AOC	GWWS	SGWWS
ENr221	55(33/22)	0.26	**0.15**	**0.07**	0.19	**0.05**
ENm010	122(63/59)	0.76	**0.04**	**0.24**	0.34	**0.15**
ENr321	108(54/54)	0.77	**5.73E-3**	**0.06**	0.35	**0.12**
ENr232	104(69/35)	0.07	**0.79**	**0.04**	0.09	**0.05**
ENr123	63(35/28)	0.43	**0.05**	**0.07**	0.42	**0.08**
ENr213	81(38/43)	1.00	**0.02**	**0.12**	0.53	**0.14**
ENr133	63(33/30)	0.88	**0.26**	**0.43**	0.74	**0.48**

Notes: We tested the overrepresentation of rare variants between the CHB (90 individuals) and JPT populations (97 individuals). For each region, the total number of variants was given, followed by the number of variants that have larger frequencies in the CHB population (the first number in the parenthesis) and the number of variants that have larger frequencies in the JPT population (the second number in the parenthesis). See notes for table 1 for abbreviation detail.

Finally, we analyzed the CHB and CHD datasets. As individuals in these two datasets are from the same population, it is expected that no difference exists between these two datasets. As expected, none region except ENr213 shows any significant difference by any test (table 7). SCollapsing and AOC detect that rare variants in the region ENr213 may distribute differently.

**Table 7:** Analyses of the ENCODE3 datasets for the CHB and CHD populations.

		Test				
Region	#variants	Collapsing	SCollapsing	AOC	GWWS	SGWWS
ENr221	53(37/16)	1.00	**0.92**	**0.44**	0.92	**0.99**
ENm010	102(67/35)	0.67	**0.12**	**0.11**	0.36	**0.42**
ENr321	101(64/37)	1.00	**0.41**	**0.83**	0.32	**0.49**
ENr232	100(78/22)	0.29	**0.59**	**0.66**	0.38	**0.43**
ENr123	51(39/12)	0.14	**0.65**	**0.68**	0.11	**0.36**
ENr213	88(60/28)	0.29	**4.32E-3**	**0.01**	0.19	**0.10**
ENr133	45(32/13)	0.83	**0.78**	**0.62**	0.92	**0.88**

Notes: We tested the overrepresentation of rare variants between the CHB (90 individuals) and CHD populations (30 individuals). For each dataset, the total number of variants was given, followed by the number of variants that have larger frequencies in the CHB population (the first number in the parenthesis) and the number of variants that have larger frequencies in the CHD population (the second number in the parenthesis). See notes for table 1 for abbreviation detail.

## Discussion

In this study, in order to improve statistical power of testing disease-associated rare variants, we proposed two alternative grouping strategies to the strategy of grouping total variants. When the proportion of neutral variants is moderate to large we show, by simulations, that both the proposed grouping strategies improve power. The applications to two real sequencing studies demonstrate the utilities of the proposed methods.

Currently, GWAS is the most widely used approach for identifying genes that are associated with complex diseases. In a typical GWAS, causative variants are usually not observable; instead, their effects are detected through nearby SNP markers, with the power depending on the strength of linkage disequilibrium (LD) between them. Thus, the pattern of LD underpins the validity and success of this indirect association approach. In sequencing studies, on the other hand, the observed genetic variants are usually hypothesized to be directly functional and are of primary interest. Furthermore, the level of LD between rare and nearby variants is usually extremely low [8]. Thus, direct association mapping is preferable for analyzing rare variants. Due to their low coverage, current genotyping platforms are inefficient for discovering rare variants. Fortunately, advances in the development of sequencing technologies [26], [27], and the recently launched 1000 genomes project [28], make sequencing huge numbers of rare variants across the entire genome a reality in the coming years.

The strategy of grouping rare variants has proven to be a powerful approach for analyzing rare variants. If potential causative variants account for the vast majority of variants, the utility of grouping total variants should be adequate, but this is unlikely to be the majority of cases in practical applications. A recent large-scale X chromosome sequencing study suggests that a large quantity of mis-sense substitutions in candidate genes exist indistinguishably in both of the case and control populations [8], indicating that a considerable proportion of mis-sense substitutions are actually background variation. Under such circumstances, the issue of distinguishing truly functional variants from background neutral variations is a major analytical challenge to this approach. Alternative grouping strategies, e.g., the two proposed in this manuscript, are warranted. The analyses of real sequencing datasets verify the superiority of the proposed methods over the existing methods.

Both of our proposed grouping methods are intuitively straightforward. In the first strategy of exhaustive grouping, by testing all subsets, we aim to identify the subset with the clearest signal. This strategy has been previously adopted in the literature of haplotype based association [29] and of gene by gene interaction studies [30]. In the second strategy of selective grouping, it is intuitive that only those variants that are overrepresented in one population could be statistically judged as the risk allele for that population. This strategy, by excluding non-informative rare variants from grouping, is expected to improve statistical power when neutral variants comprise a substantial large proportion of the total variants. We have demonstrated, through simulations, that our proposed alternative grouping methods partially solve the problem created by the total grouping strategy which ignores the noise of background population variation.

In the selective grouping, two sets *G_c_* and *G_u_* are formed. While the *G_c_* part may represent potentially causative variants, *G_u_* part is believed to represent background variation. However, including *G_u_* part into analysis could provide a two-sided test of whether variants are associated with the phenotype rather than a one-sided test of whether variants enrich in a particular population, i.e., cases. This broader hypothesis makes it comparable to compare between tests with various grouping strategies. The *G_u_* part could also be informative in the presence of beneficial mutations, i.e., those mutations which reduce the risk to the disease.

For a set of *L* rare variants, the time complexity for methods with exhaustive grouping is O(2*^L^*), while that for methods without exhaustive groupings is only O(1). The computation time for exhaustive grouping increases exponentially with the number of variants, and is prohibitive for analyzing several dozens to hundreds of variants. To circumvent this computation issue, we have developed a step-wise algorithm to search for the minimal p-value over the set, the time complexity of which is O(*L^2^*). Alternatively, the proposed selective grouping method is computationally effective, without additional increase in time complexity compared to total grouping. In practical applications where huge numbers of rare variants may be involved, e.g., exome studies, the selective grouping method is thus recommended, and exhaustive grouping should be regarded as a tool to verify significant results.

Our simulations show that statistical power of testing association decreases as gene elongates, though the proportion of causative variants is fixed. To understand this phenomenon, gene heritability must be taken into consideration, which was always fixed at 0.5% as gene length varied in our simulations. Longer gene introduces more noise, which tends to reduce power. Though meanwhile it introduces a greater number of causative variants as well, the phenotypic effect of these causative variants, that is, gene heritability, remains unchanged. In other words, the per-variant effect gets smaller with longer gene. Consequently, though signal-to-noise ratio is fixed, the power would still tend to decrease as gene elongates. This trend of power with gene length (or number of variants) was also observed in previous studies [16], [17].

The problem of population stratification by admixture of different ethnic populations is a serious concern for association studies [31], [32], [33] with both common and rare variants. Strictly matched case-control pairs, e.g., affected case and its unaffected sib, provide the most reliable guard against population stratification. However, recruiting such samples is costly and difficult. For association studies with common variants, spurious associations can be controlled at the population level, towards which a variety of approaches have been proposed [34], [35], [36]. For studies with rare variants, on the other hand, little is known on the magnitude of the impact of population stratification and on the performance of existing correction approaches. We therefore restricted our analyses to homogeneous populations, but the issue of population stratification deserves further investigation.

In this study, we used a MAF cutoff 0.01 for a rare variant, but other cutoffs could be used as well. In our simulation studies we show that our proposed strategies could improve power when the proportion of causative variants is low to modest, while the power improvement is little or even negative when the proportion is large. Thus, the proper test for a particular study should be chosen according to the knowledge on the distribution of potential causative variants. In most practical applications where there is no knowledge on the potential causative variants, cross-validation of the results from various tests is warranted.

Statistical methods derived not from classical statistical theories, like the ones proposed in this manuscript, may be biased. The biasness could be evaluated by simulation studies, which showed that on one hand, the power rate of the proposed tests was equal to the significance level under the null hypothesis. On the other hand, the power rate was an increasing function of locus effect and/or proportion of causative variants so that it would excess the significance level under the alternative hypothesis. These two aspects of power function under the null and alternative hypothesis together indicated that the proposed tests were unbiased.

Despite the methods proposed in this manuscript, analyzing associations between rare variants and complex diseases remains quite challenging. When neutral variants comprise a large proportion of the total variants, the selective grouping is still low-powered, and more specialized approaches are expected to emerge. In the study of Tarpey et al. [8], three new genes were identified for mental retardation. From a statistical point of view, however, none of the three genes were statistically significant. Rather, the authors compared amino acids with their orthologs from other species, and ranked the importance of amino acids according to their conservation scores. This approach holds promise for further methodological development and might provide another tool for performing powerful association tests by considering biological or evolutionary information.

## Supporting Information

Appendix S1 The appendix that describes the process of demographic model.
(0.09 MB DOC)

Figure S1 Fitness of simulated variants to experimental variants. Legend: Sequence data of European population from the ENCODE3 project were used to estimate demographic model. Data of 119 individuals (238 haplotypes) on 7 genomic regions were available. After filtering out variants with missing genotypes, a total of 83 gene-coding variants and 953 non-coding (neutral) variants were used for analysis, corresponding to 5.3 kb and 66.7 kb sequence sites respectively. A: the fitness of simulated allele frequencies to experimental data on neutral variants; B: the fitness of simulated allele frequencies to experimental data on gene-coding variants.
(0.27 MB TIF)

Table S1 Phenotypic models versus encoding schemes
(0.07 MB DOC)
