# Supplementary material for: Efficient Utilization of Rare Variants for Detection of Disease-Related Genomic Regions
Source: PLoS One. 2010 Dec 10;5(12):e14288. doi: 10.1371/journal.pone.0014288 (PMC3000820; doi:10.1371/journal.pone.0014288)
Supplement: Appendix S1 — The appendix that describes the process of demographic model. (0.09 MB DOC) [file pone.0014288.s001.doc]

**Appendix**

Following [1], let $\varphi(q,t|p,n)$ be the probability density that the allele frequency in the *t*th generation is *q* given that its frequency in the initial population (with size *n*) is *p*. For a constant population size *N*_0_, $\varphi$ is given by [1,2]

$\varphi(q,t|p,N_{0})=\sum_{i=1}^{\infty} \frac{(2i+1)(1-(1-2p)^{2})}{i(i+1)}\cdot C_{i-1}^{3/2}(1-2p)\cdot C_{i-1}^{3/2}(1-2q)\cdot e^{-\frac{i(i+1)}{4N_{0}}t}$,

where $C_{i-1}^{3/2}$ is the Gegenbauer polynomial with $\lambda=3/2$. For a exponentially growing population with $N_{t}=N_{0}\cdot e^{\gamma t}$, $\varphi$ has a solution by replacing *t* with an effective time $t^{'}=(1-e^{-\gamma t})/\gamma$, $\varphi(q,t^{'}|p,N_{0})=\varphi(q,(1-e^{-\gamma t})/\gamma|p,N_{0}).$

The probability density of allele frequency in the present population (*t* = *T*) is the sum of contributions of mutations that originated from the ancestral population before the bottleneck and that originated from the expanding population after the bottleneck

$$f(q)=4N_{1}\mu\sum_{i=1}^{2N_{b}-1} \frac{1}{i}\cdot\varphi(q,\frac{1-e^{-\gamma T}}{\gamma}|\frac{i}{2N_{b}},N_{b})+2N_{b}\mu\sum_{t=1}^{T} e^{\gamma t}\varphi(q,\frac{1-e^{-(T-t)\gamma}}{\gamma}|\frac{1}{2N_{b}e^{\gamma t}},N_{b}e^{\gamma t}),$$

where $\mu$ is the per site mutation rate. Here we set $\mu=1.8\text{E}-8$ [3].

For an observed sequence sample with *N_s_* chromosomes, the site spectrum frequency is defined as the $\frac{N_{s}}{2}+1$ dimensional vector $\mathbf{r}=(r_{0},r_{1},...,r_{\frac{N_{s}}{2}})$, where *r_i_* is the number of sites at which the minor allele is observed *i* times. By the above demographic model, the probability that the minor allele is observed *i* ($1\leq i<\frac{N_{s}}{2}$) times is given by

$$F_{i}=\int_{0}^{1} \left(_{i}^{N_{s}}\right)(q^{i}(1-q)^{N_{s}-i}+q^{N_{s}-i}(1-q)^{i})f(q)dq,$$

and $F_{\frac{N_{s}}{2}}=\int_{0}^{1} \left(_{\frac{N_{s}}{2}}^{N_{s}}\right)(q(1-q))^{\frac{N_{s}}{2}}f(q)dq.$

The probability of observing monomorphic site is given by $F_{0}=1-\sum_{i=1}^{N_{s}/2} F_{i}.$

Assuming the independence between sites, the likelihood of the observed site frequency spectrum data is given by

$$L(N_{1},N_{b},T,\gamma)=p(\mathbf{r}|N_{1},N_{b},T,\gamma)=\prod_{i=0}^{N_{s}/2} F_{i}^{r_{i}}.$$

Given the observed sequence data, the model parameters $\theta=[N_{1},N_{b},T,\gamma]$ will be estimated by maximizing the likelihood function *L*. To estimate $\theta$, we analyze the real sequence dataset produced by the ENCODE3 project [4]. In the ENCODE3 project, ten genomic regions each comprising 100 kb sites were sequenced in many populations. Data (released on March 14^th^ 2008) were downloaded from the project ftp site (ftp://ftp.hgsc.bcm.tmc.edu/pub/data/HapMap3-ENCODE/ENCODE3/ENCODE3v1/). We use sequence data from the European population, which consists of 238 chromosomes from 119 individuals. Data from 7 genomic regions are available for analysis. Of the 700 kb genomic regions sequenced, 58.2 kb are gene-coding regions and the remaining 641.8 kb are non-coding regions. A total of 10,076 variants were reported in the raw dataset, of which 911 are in gene-coding regions. For quality control, we select variants that were successfully sequenced in all individuals, resulting in 83 gene-coding variants and 953 non-coding variants for analysis. These variants correspond approximately to 66.7 (641.8 x 953/9165) kb and 5.3 (58.2 x 83/911) kb sites sequenced in non-coding and coding regions, respectively. We use only non-coding variants to estimate the demographic model. The parameters estimated by maximum likelihood estimation are *N*_1_=20,000, *N_b_* =14,000, *T* =3,300, and $\gamma$=0.001. *N*_1_ and *N_b_* are larger than a previous report by two fold [5]. Given an average 20 years per human generation, the estimated *T* implies that the bottleneck occurred ~66,000 years ago (66 kya). This estimation is consistent with the “out-of-Africa” event which presumes that the European branch was expanded from Africa ~80-40 kya. The estimated effective size of today’s population is ~380,000, much larger than that reported in [5] (20,000), but smaller than that reported in [1] (900,000). For neutral variants the estimated allele frequencies from simulations matched quite well with experimental data (Figure S1, A). Due to the limited number of non-synonymous variants in this dataset, the strength of natural selection could not be evaluated. Instead, using a model with neutral selection, the frequency predicted by simulation for alleles in gene coding regions matched quite well with the experimental data from the ENCODE3 project (Figure S1, B). This may be because the effect of selection was too weak to be detected by the limited number of non-synonymous variants available.

**REFERENCES**

1. Kryukov GV, Shpunt A, Stamatoyannopoulos JA, Sunyaev SR (2009) Power of deep, all-exon resequencing for discovery of human trait genes. Proc Natl Acad Sci U S A 106: 3871-3876.

2. Williamson SH, Hernandez R, Fledel-Alon A, Zhu L, Nielsen R, et al. (2005) Simultaneous inference of selection and population growth from patterns of variation in the human genome. Proc Natl Acad Sci U S A 102: 7882-7887.

3. Sunyaev S, Ramensky V, Koch I, Lathe W, 3rd, Kondrashov AS, et al. (2001) Prediction of deleterious human alleles. Hum Mol Genet 10: 591-597.

4. Birney E, Stamatoyannopoulos JA, Dutta A, Guigo R, Gingeras TR, et al. (2007) Identification and analysis of functional elements in 1% of the human genome by the ENCODE pilot project. Nature 447: 799-816.

5. Marth GT, Czabarka E, Murvai J, Sherry ST (2004) The allele frequency spectrum in genome-wide human variation data reveals signals of differential demographic history in three large world populations. Genetics 166: 351-372.

**FIGURE LEGEND**

Figure S1. Fitness of simulated variants to experimental variants

Sequence data of European population from the ENCODE3 project were used to estimate demographic model. Data of 119 individuals (238 haplotypes) on 7 genomic regions were available. After filtering out variants with missing genotypes, a total of 83 gene-coding variants and 953 non-coding (neutral) variants were used for analysis, corresponding to 5.3kb and 66.7kb sequence sites respectively. A: the fitness of simulated allele frequencies to experimental data on neutral variants; B: the fitness of simulated allele frequencies to experimental data on gene-coding variants.
