# Supplementary figures and images for: Efficient Utilization of Rare Variants for Detection of Disease-Related Genomic Regions
Source: PLoS One. 2010 Dec 10;5(12):e14288. doi: 10.1371/journal.pone.0014288 (PMC3000820; doi:10.1371/journal.pone.0014288)

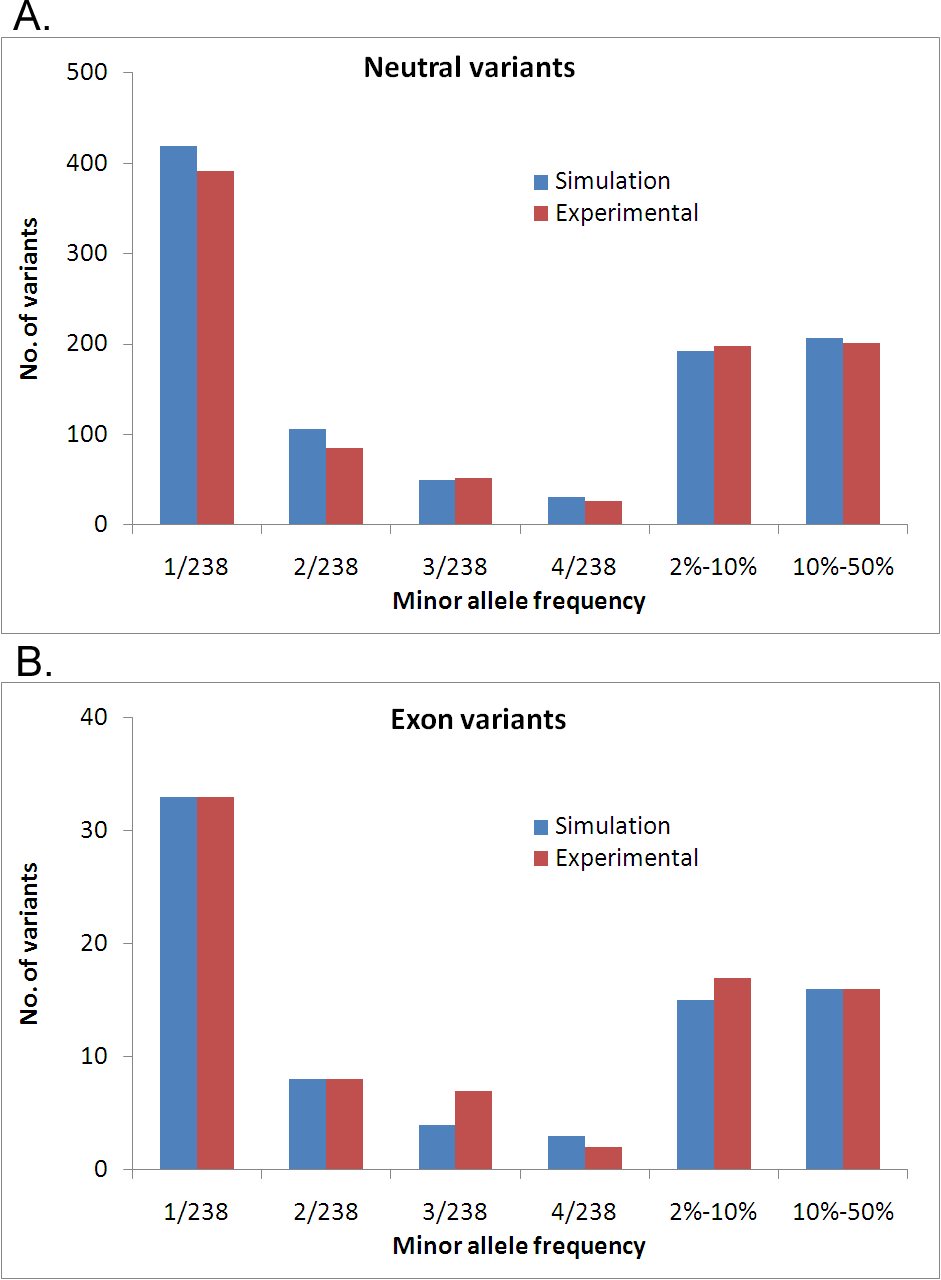

Supplement: Figure S1 — Fitness of simulated variants to experimental variants. Legend: Sequence data of European population from the ENCODE3 project were used to estimate demographic model. Data of 119 individuals (238 haplotypes) on 7 genomic regions were available. After filtering out variants with missing genotypes, a total of 83 gene-coding variants and 953 non-coding (neutral) variants were used for analysis, corresponding to 5.3 kb and 66.7 kb sequence sites respectively. A: the fitness of simulated allele frequencies to experimental data on neutral variants; B: the fitness of simulated allele frequencies to experimental data on gene-coding variants. (0.27 MB TIF) [file pone.0014288.s002.tif]
