# Supplementary material for: Efficient Utilization of Rare Variants for Detection of Disease-Related Genomic Regions
Source: PLoS One. 2010 Dec 10;5(12):e14288. doi: 10.1371/journal.pone.0014288 (PMC3000820; doi:10.1371/journal.pone.0014288)
Supplement: Table S1 — Phenotypic models versus encoding schemes (0.07 MB DOC) [file pone.0014288.s003.doc]

Table S1. Phenotypic models versus encoding schemes

|  | Phenotypic model (*x*) | | |
| --- | --- | --- | --- |
| Test (***X***) | Model 1 | Model 2 | Model 3 |
| Collapsing | $x_{i}=\sum_{l=1}^{L_{c}} g_{il}$ | $x_{i}=\left\{_{0\text{ otherwise}}^{1\text{ causative present}}\right\}$ $x_{i}=\sum_{l=1}^{L_{c}} \frac{g_{il}}{w_{l}},$ $w_{l}=\sqrt{p_{l}(1-p_{l})}$ $X_{i}=\left\{_{0\text{ otherwise}}^{1\text{ rare present}}\right.$ | |
| GWWS | $X_{i}=\sum_{l=1}^{L} \frac{I_{il}}{\hat{w}_{l}},$ $x_{i}=\sum_{l=1}^{L_{c}} g_{il}$ $x_{i}=\left\{_{0\text{ otherwise}}^{1\text{ causative present}}\right\}$ | $\hat{w}_{l}=\sqrt{n\cdot q_{l}(1-q_{l})}$ | $x_{i}=\sum_{l=1}^{L_{c}} \frac{g_{il}}{w_{l}},$ $w_{l}=\sqrt{p_{l}(1-p_{l})}$ |

Notes: Three phenotypic models were simulated. Under each model, two tests Collapsing and GWWS with different genotypic encoding schemes were examined.
